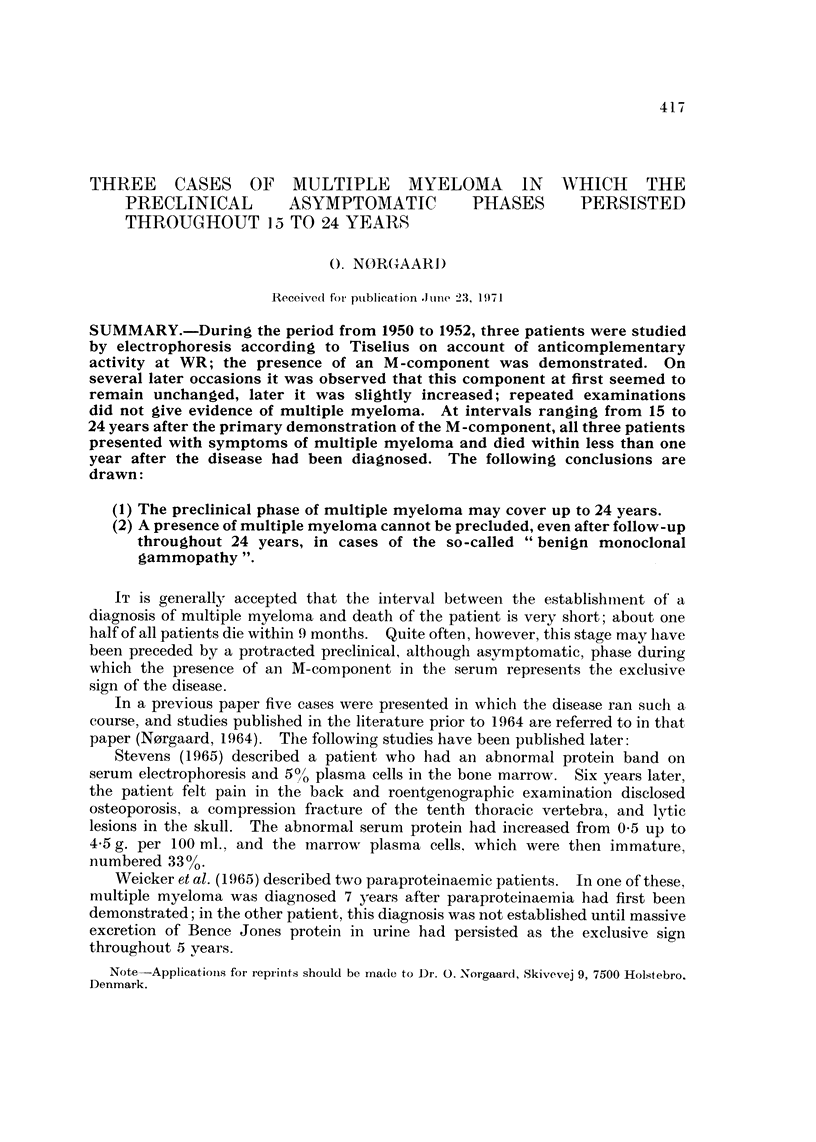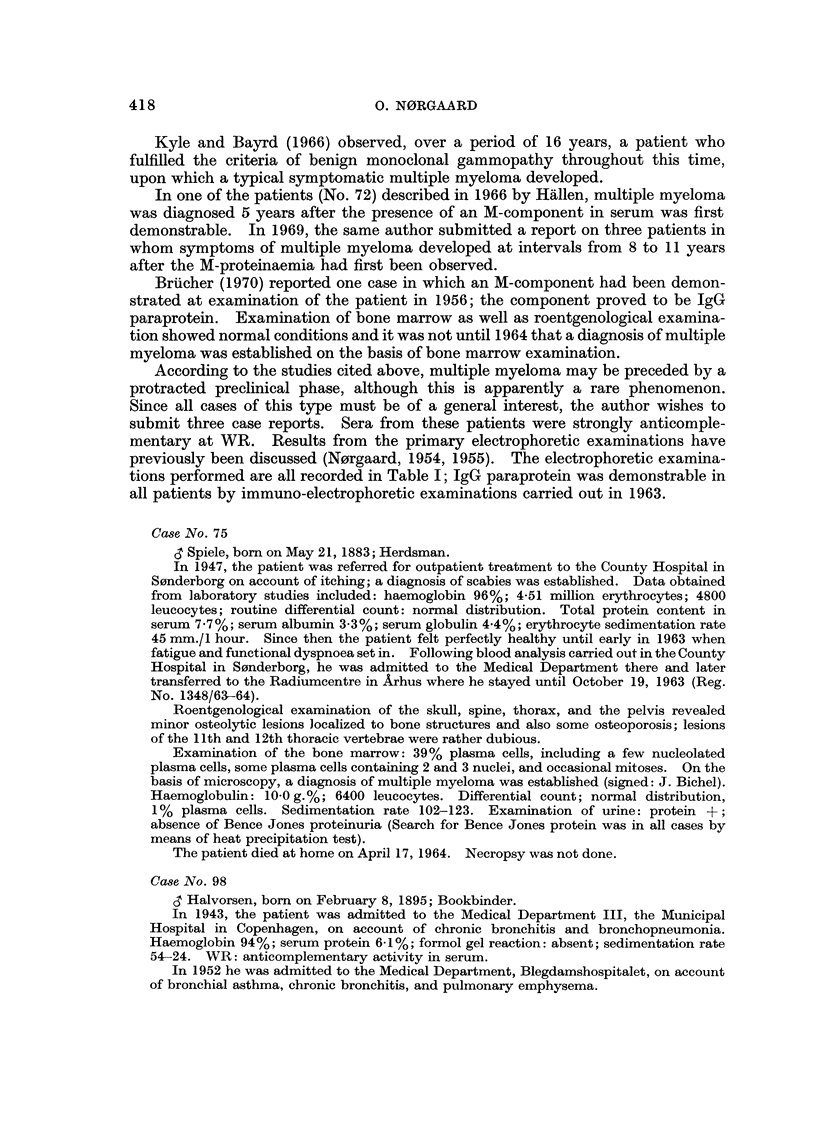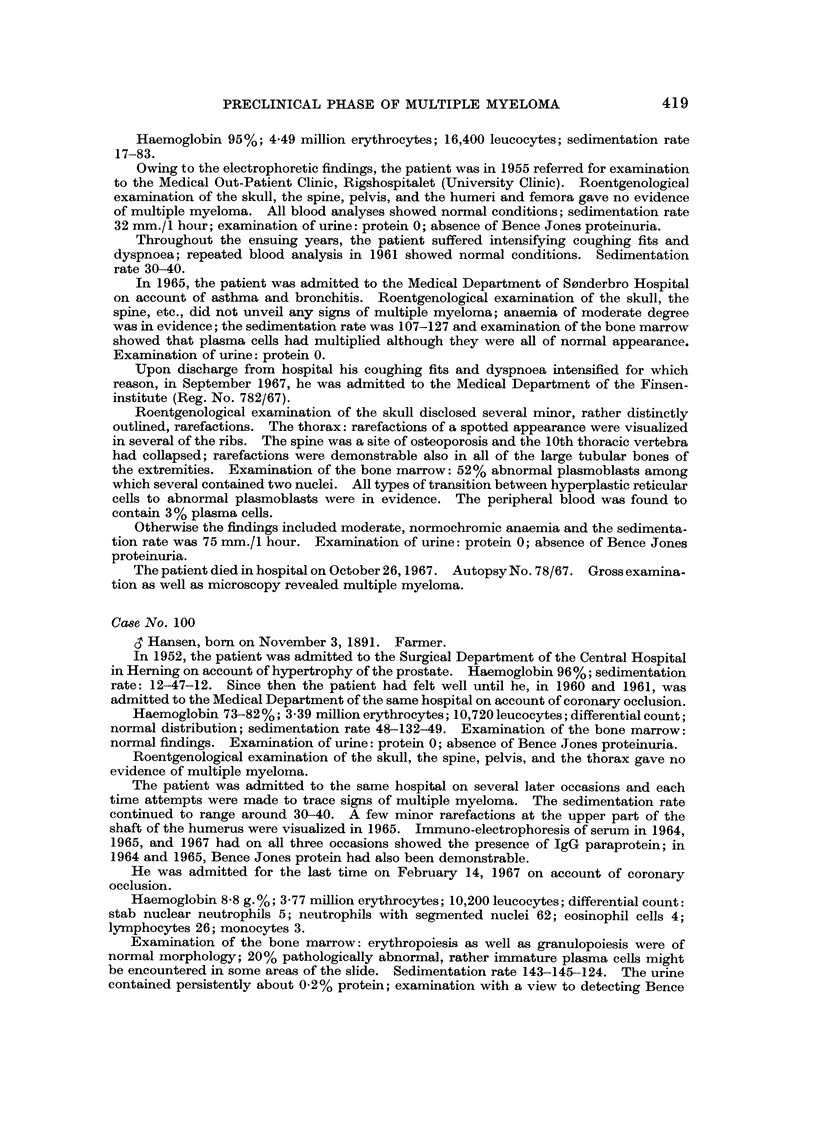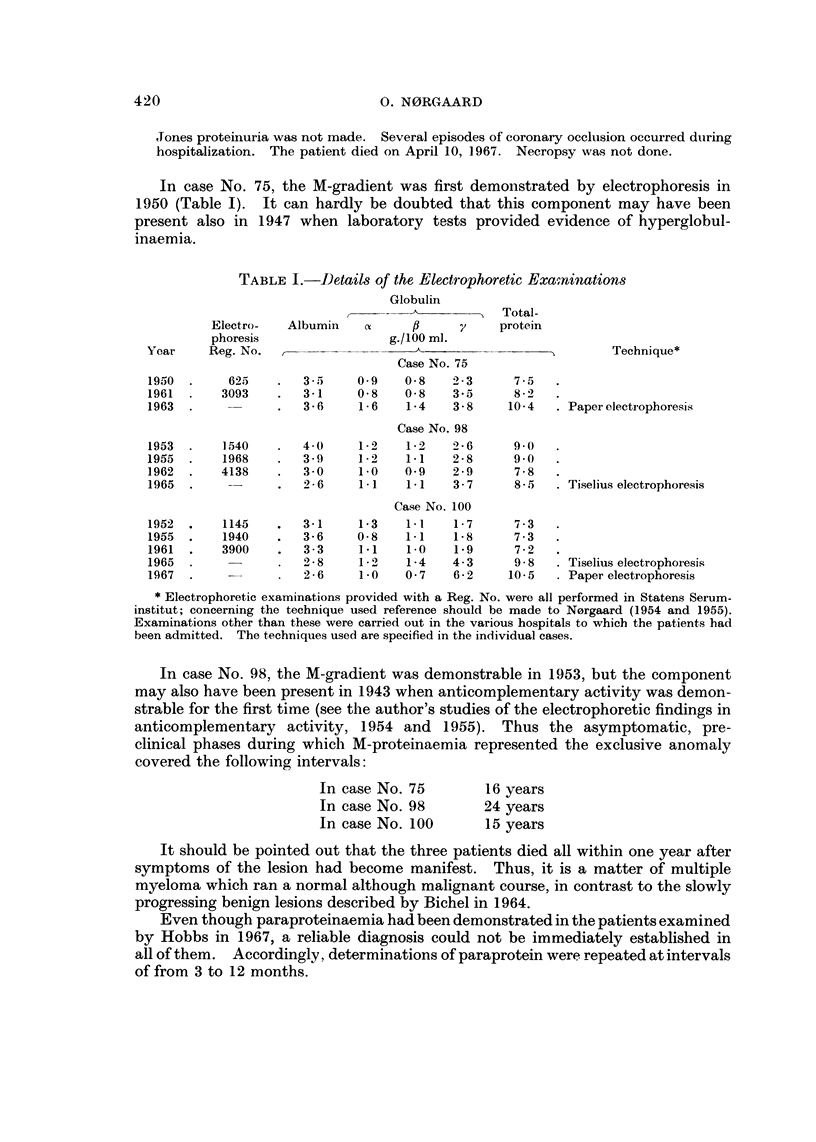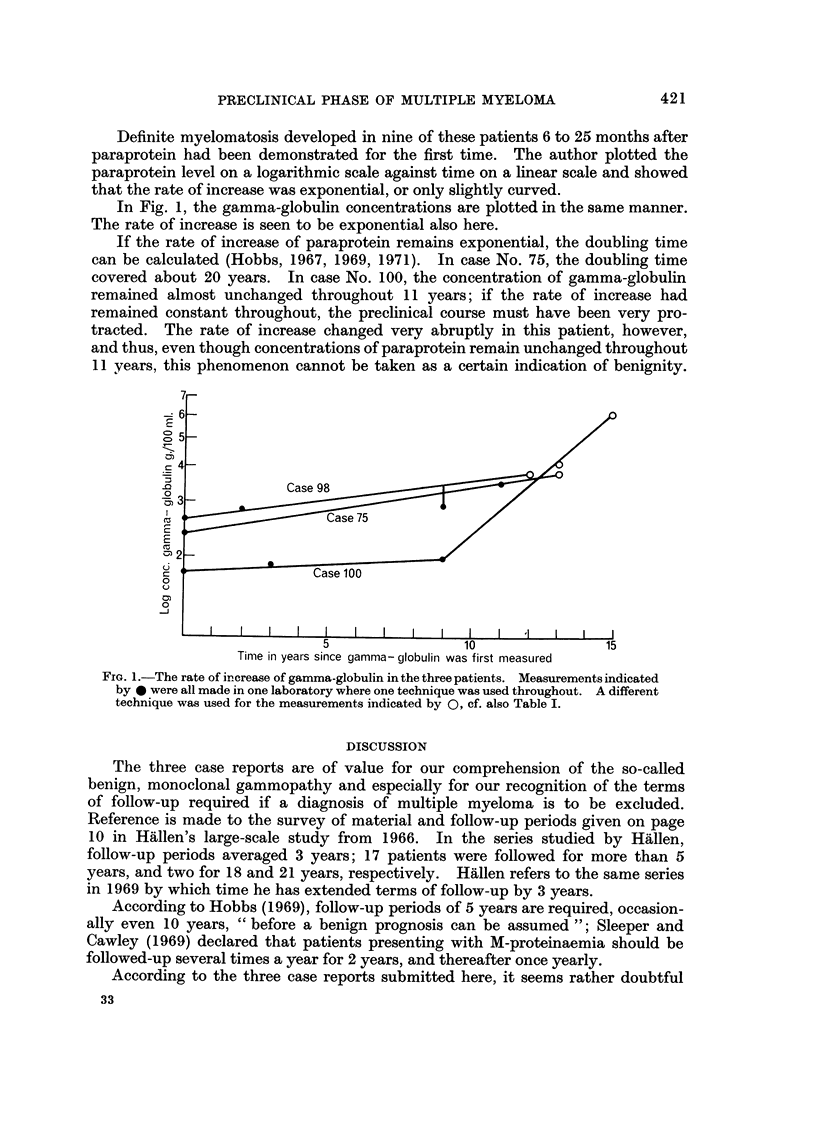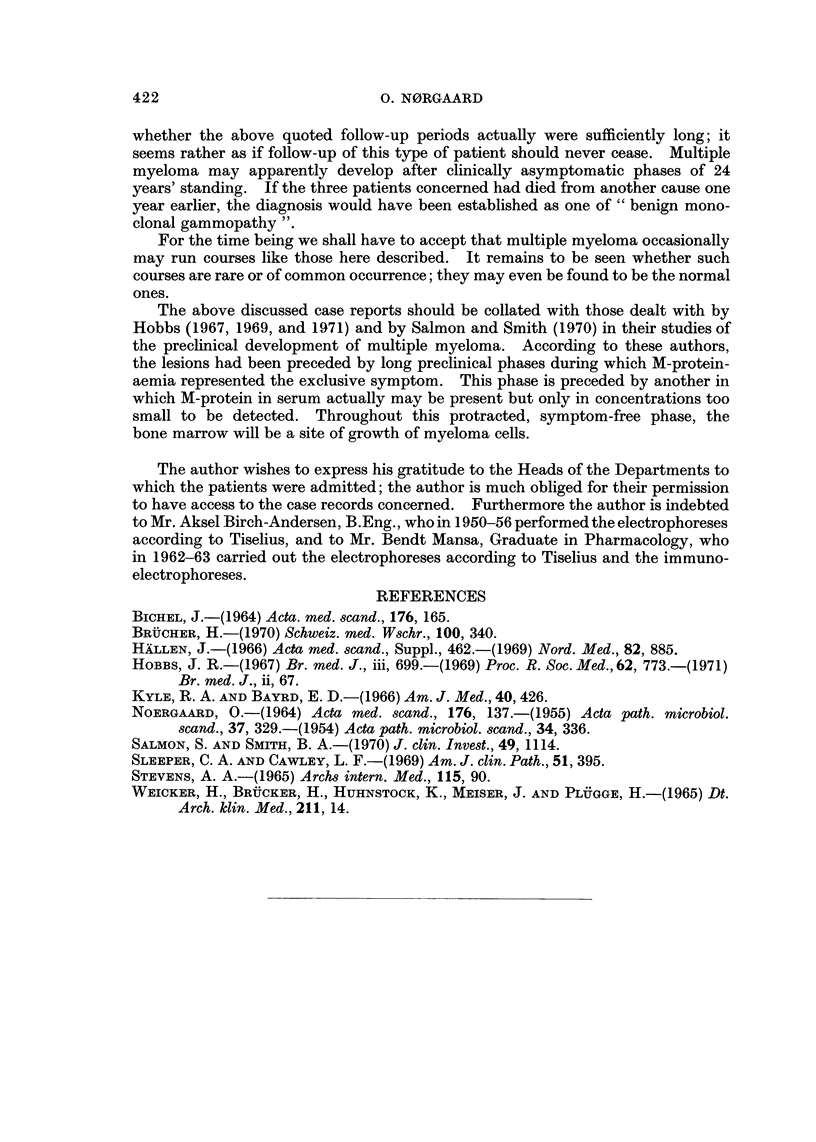# Three Cases of Multiple Myeloma in which the Preclinical Asymptomatic Phases Persisted Throughout 15 to 24 Years

**DOI:** 10.1038/bjc.1971.53

**Published:** 1971-09

**Authors:** O. Nøorgaard

## Abstract

During the period from 1950 to 1952, three patients were studied by electrophoresis according to Tiselius on account of anticomplementary activity at WR; the presence of an M-component was demonstrated. On several later occasions it was observed that this component at first seemed to remain unchanged, later it was slightly increased; repeated examinations did not give evidence of multiple myeloma. At intervals ranging from 15 to 24 years after the primary demonstration of the M-component, all three patients presented with symptoms of multiple myeloma and died within less than one year after the disease had been diagnosed. The following conclusions are drawn:

(1) The preclinical phase of multiple myeloma may cover up to 24 years.

(2) A presence of multiple myeloma cannot be precluded, even after follow-up throughout 24 years, in cases of the so-called “benign monoclonal gammopathy”.


### THREE CASES OF MULTIPLE MYELOMA IN WHICH THE PRECLINICAL ASYMPTOMATIC PHASES PERSISTED THROUGHOUT 15 TO 24 YEARS

O. NØRGAARD

Received for publication June 23, 1971

**SUMMARY.—During the period from 1950 to 1952, three patients were studied by electrophoresis according to Tiselius on account of anticomplementary activity at WR; the presence of an M-component was demonstrated. On several later occasions it was observed that this component at first seemed to remain unchanged, later it was slightly increased; repeated examinations did not give evidence of multiple myeloma. At intervals ranging from 15 to 24 years after the primary demonstration of the M-component, all three patients presented with symptoms of multiple myeloma and died within less than one year after the disease had been diagnosed. The following conclusions are drawn:**

**(1) The preclinical phase of multiple myeloma may cover up to 24 years.**
**(2) A presence of multiple myeloma cannot be precluded, even after follow-up throughout 24 years, in cases of the so-called " benign monoclonal gammopathy ".**

It is generally accepted that the interval between the establishment of a diagnosis of multiple myeloma and death of the patient is very short; about one half of all patients die within 9 months. Quite often, however, this stage may have been preceded by a protracted preclinical, although asymptomatic, phase during which the presence of an M-component in the serum represents the exclusive sign of the disease.

In a previous paper five cases were presented in which the disease ran such a course, and studies published in the literature prior to 1964 are referred to in that paper (Nørgaard, 1964). The following studies have been published later:

Stevens (1965) described a patient who had an abnormal protein band on serum electrophoresis and 5% plasma cells in the bone marrow. Six years later, the patient felt pain in the back and roentgenographic examination disclosed osteoporosis, a compression fracture of the tenth thoracic vertebra, and lytic lesions in the skull. The abnormal serum protein had increased from 0·5 up to 4·5 g. per 100 ml., and the marrow plasma cells, which were then immature, numbered 33%.

Weicker *et al.* (1965) described two paraproteinaemic patients. In one of these, multiple myeloma was diagnosed 7 years after paraproteinaemia had first been demonstrated; in the other patient, this diagnosis was not established until massive excretion of Bence Jones protein in urine had persisted as the exclusive sign throughout 5 years.

Note—Applications for reprints should be made to Dr. O. Norgaard, Skivevej 9, 7500 Holstebro. Denmark.

Kyle and Bayrd (1966) observed, over a period of 16 years, a patient who fulfilled the criteria of benign monoclonal gammopathy throughout this time, upon which a typical symptomatic multiple myeloma developed.

In one of the patients (No. 72) described in 1966 by Hällen, multiple myeloma was diagnosed 5 years after the presence of an M-component in serum was first demonstrable. In 1969, the same author submitted a report on three patients in whom symptoms of multiple myeloma developed at intervals from 8 to 11 years after the M-proteinaemia had first been observed.

Brücher (1970) reported one case in which an M-component had been demonstrated at examination of the patient in 1956; the component proved to be IgG paraprotein. Examination of bone marrow as well as roentgenological examination showed normal conditions and it was not until 1964 that a diagnosis of multiple myeloma was established on the basis of bone marrow examination.

According to the studies cited above, multiple myeloma may be preceded by a protracted preclinical phase, although this is apparently a rare phenomenon. Since all cases of this type must be of a general interest, the author wishes to submit three case reports. Sera from these patients were strongly anticomplementary at WR. Results from the primary electrophoretic examinations have previously been discussed (Nørgaard, 1954, 1955). The electrophoretic examinations performed are all recorded in Table I; IgG paraprotein was demonstrable in all patients by immuno-electrophoretic examinations carried out in 1963.

*Case No.* 75

♂ Spiele, born on May 21, 1883; Herdsman.

In 1947, the patient was referred for outpatient treatment to the County Hospital in Sønderborg on account of itching; a diagnosis of scabies was established. Data obtained from laboratory studies included: haemoglobin 96%; 4·51 million erythrocytes; 4800 leucocytes; routine differential count: normal distribution. Total protein content in serum 7·7%; serum albumin 3·3%; serum globulin 4·4%; erythrocyte sedimentation rate 45 mm./1 hour. Since then the patient felt perfectly healthy until early in 1963 when fatigue and functional dyspnoea set in. Following blood analysis carried out in the County Hospital in Sønderborg, he was admitted to the Medical Department there and later transferred to the Radiumcentre in Århus where he stayed until October 19, 1963 (Reg. No. 1348/63–64).

Roentgenological examination of the skull, spine, thorax, and the pelvis revealed minor osteolytic lesions localized to bone structures and also some osteoporosis; lesions of the 11th and 12th thoracic vertebrae were rather dubious.

Examination of the bone marrow: 39% plasma cells, including a few nucleolated plasma cells, some plasma cells containing 2 and 3 nuclei, and occasional mitoses. On the basis of microscopy, a diagnosis of multiple myeloma was established (signed: J. Bichel). Haemoglobulin: 10·0 g.%; 6400 leucocytes. Differential count; normal distribution, 1% plasma cells. Sedimentation rate 102–123. Examination of urine: protein +; absence of Bence Jones proteinuria (Search for Bence Jones protein was in all cases by means of heat precipitation test).

The patient died at home on April 17, 1964. Necropsy was not done.

*Case No.* 98

♂ Halvorsen, born on February 8, 1895; Bookbinder.

In 1943, the patient was admitted to the Medical Department III, the Municipal Hospital in Copenhagen, on account of chronic bronchitis and bronchopneumonia. Haemoglobin 94%; serum protein 6·1%; formol gel reaction: absent; sedimentation rate 54–24. WR: anticomplementary activity in serum.

In 1952 he was admitted to the Medical Department, Blegdamshospitalet, on account of bronchial asthma, chronic bronchitis, and pulmonary emphysema.

Haemoglobin 95%; 4·49 million erythrocytes; 16,400 leucocytes; sedimentation rate 17–83.

Owing to the electrophoretic findings, the patient was in 1955 referred for examination to the Medical Out-Patient Clinic, Rigshospitalet (University Clinic). Roentgenological examination of the skull, the spine, pelvis, and the humeri and femora gave no evidence of multiple myeloma. All blood analyses showed normal conditions; sedimentation rate 32 mm./1 hour; examination of urine: protein 0; absence of Bence Jones proteinuria.

Throughout the ensuing years, the patient suffered intensifying coughing fits and dyspnoea; repeated blood analysis in 1961 showed normal conditions. Sedimentation rate 30–40.

In 1965, the patient was admitted to the Medical Department of Sønderbro Hospital on account of asthma and bronchitis. Roentgenological examination of the skull, the spine, etc., did not unveil any signs of multiple myeloma; anaemia of moderate degree was in evidence; the sedimentation rate was 107–127 and examination of the bone marrow showed that plasma cells had multiplied although they were all of normal appearance. Examination of urine: protein 0.

Upon discharge from hospital his coughing fits and dyspnoea intensified for which reason, in September 1967, he was admitted to the Medical Department of the Finsen-institute (Reg. No. 782/67).

Roentgenological examination of the skull disclosed several minor, rather distinctly outlined, rarefactions. The thorax: rarefactions of a spotted appearance were visualized in several of the ribs. The spine was a site of osteoporosis and the 10th thoracic vertebra had collapsed; rarefactions were demonstrable also in all of the large tubular bones of the extremities. Examination of the bone marrow: 52% abnormal plasmoblasts among which several contained two nuclei. All types of transition between hyperplastic reticular cells to abnormal plasmoblasts were in evidence. The peripheral blood was found to contain 3% plasma cells.

Otherwise the findings included moderate, normochromic anaemia and the sedimentation rate was 75 mm./1 hour. Examination of urine: protein 0; absence of Bence Jones proteinuria.

The patient died in hospital on October 26, 1967. Autopsy No. 78/67. Gross examination as well as microscopy revealed multiple myeloma.

*Case No.* 100

♂ Hansen, born on November 3, 1891. Farmer.

In 1952, the patient was admitted to the Surgical Department of the Central Hospital in Herning on account of hypertrophy of the prostate. Haemoglobin 96%; sedimentation rate: 12–47–12. Since then the patient had felt well until he, in 1960 and 1961, was admitted to the Medical Department of the same hospital on account of coronary occlusion.

Haemoglobin 73–82%; 3·39 million erythrocytes; 10,720 leucocytes; differential count; normal distribution; sedimentation rate 48–132–49. Examination of the bone marrow: normal findings. Examination of urine: protein 0; absence of Bence Jones proteinuria.

Roentgenological examination of the skull, the spine, pelvis, and the thorax gave no evidence of multiple myeloma.

The patient was admitted to the same hospital on several later occasions and each time attempts were made to trace signs of multiple myeloma. The sedimentation rate continued to range around 30–40. A few minor rarefactions at the upper part of the shaft of the humerus were visualized in 1965. Immuno-electrophoresis of serum in 1964, 1965, and 1967 had on all three occasions showed the presence of IgG paraprotein; in 1964 and 1965, Bence Jones protein had also been demonstrable.

He was admitted for the last time on February 14, 1967 on account of coronary occlusion.

Haemoglobin 8·8 g.%; 3·77 million erythrocytes; 10,200 leucocytes; differential count: stab nuclear neutrophils 5; neutrophils with segmented nuclei 62; eosinophil cells 4; lymphocytes 26; monocytes 3.

Examination of the bone marrow: erythropoiesis as well as granulopoiesis were of normal morphology; 20% pathologically abnormal, rather immature plasma cells might be encountered in some areas of the slide. Sedimentation rate 143–145–124. The urine contained persistently about 0·2% protein; examination with a view to detecting Bence

Jones proteinuria was not made. Several episodes of coronary occlusion occurred during hospitalization. The patient died on April 10, 1967. Necropsy was not done.

In case No. 75, the M-gradient was first demonstrated by electrophoresis in 1950 (Table I). It can hardly be doubted that this component may have been present also in 1947 when laboratory tests provided evidence of hyperglobulinaemia.

TABLE I.—*Details of the Electrophoretic Examinations*

| Year | Electrophoresis Reg. No. | Albumin | Globulin α | Globulin β | Globulin γ | Total-protein | Technique* |
|---|---|---|---|---|---|---|---|
| | | g./100 ml. | | | | | |
| | | Case No. 75 | | | | | |
| 1950 | 625 | 3·5 | 0·9 | 0·8 | 2·3 | 7·5 | |
| 1961 | 3093 | 3·1 | 0·8 | 0·8 | 3·5 | 8·2 | |
| 1963 | — | 3·6 | 1·6 | 1·4 | 3·8 | 10·4 | Paper electrophoresis |
| | | Case No. 98 | | | | | |
| 1953 | 1540 | 4·0 | 1·2 | 1·2 | 2·6 | 9·0 | |
| 1955 | 1968 | 3·9 | 1·2 | 1·1 | 2·8 | 9·0 | |
| 1962 | 4138 | 3·0 | 1·0 | 0·9 | 2·9 | 7·8 | |
| 1965 | — | 2·6 | 1·1 | 1·1 | 3·7 | 8·5 | Tiselius electrophoresis |
| | | Case No. 100 | | | | | |
| 1952 | 1145 | 3·1 | 1·3 | 1·1 | 1·7 | 7·3 | |
| 1955 | 1940 | 3·6 | 0·8 | 1·1 | 1·8 | 7·3 | |
| 1961 | 3900 | 3·3 | 1·1 | 1·0 | 1·9 | 7·2 | |
| 1965 | — | 2·8 | 1·2 | 1·4 | 4·3 | 9·8 | Tiselius electrophoresis |
| 1967 | — | 2·6 | 1·0 | 0·7 | 6·2 | 10·5 | Paper electrophoresis |

* Electrophoretic examinations provided with a Reg. No. were all performed in Statens Seruminstitut; concerning the technique used reference should be made to Nørgaard (1954 and 1955). Examinations other than these were carried out in the various hospitals to which the patients had been admitted. The techniques used are specified in the individual cases.

In case No. 98, the M-gradient was demonstrable in 1953, but the component may also have been present in 1943 when anticomplementary activity was demonstrable for the first time (see the author's studies of the electrophoretic findings in anticomplementary activity, 1954 and 1955). Thus the asymptomatic, preclinical phases during which M-proteinaemia represented the exclusive anomaly covered the following intervals:

| | |
|---|---|
| In case No. 75 | 16 years |
| In case No. 98 | 24 years |
| In case No. 100 | 15 years |

It should be pointed out that the three patients died all within one year after symptoms of the lesion had become manifest. Thus, it is a matter of multiple myeloma which ran a normal although malignant course, in contrast to the slowly progressing benign lesions described by Bichel in 1964.

Even though paraproteinaemia had been demonstrated in the patients examined by Hobbs in 1967, a reliable diagnosis could not be immediately established in all of them. Accordingly, determinations of paraprotein were repeated at intervals of from 3 to 12 months.

Definite myelomatosis developed in nine of these patients 6 to 25 months after paraprotein had been demonstrated for the first time. The author plotted the paraprotein level on a logarithmic scale against time on a linear scale and showed that the rate of increase was exponential, or only slightly curved.

In Fig. 1, the gamma-globulin concentrations are plotted in the same manner. The rate of increase is seen to be exponential also here.

If the rate of increase of paraprotein remains exponential, the doubling time can be calculated (Hobbs, 1967, 1969, 1971). In case No. 75, the doubling time covered about 20 years. In case No. 100, the concentration of gamma-globulin remained almost unchanged throughout 11 years; if the rate of increase had remained constant throughout, the preclinical course must have been very protracted. The rate of increase changed very abruptly in this patient, however, and thus, even though concentrations of paraprotein remain unchanged throughout 11 years, this phenomenon cannot be taken as a certain indication of benignity.

FIG. 1.—The rate of increase of gamma-globulin in the three patients. Measurements indicated by ● were all made in one laboratory where one technique was used throughout. A different technique was used for the measurements indicated by ○, cf. also Table I.

### DISCUSSION

The three case reports are of value for our comprehension of the so-called benign, monoclonal gammopathy and especially for our recognition of the terms of follow-up required if a diagnosis of multiple myeloma is to be excluded. Reference is made to the survey of material and follow-up periods given on page 10 in Hällen's large-scale study from 1966. In the series studied by Hällen, follow-up periods averaged 3 years; 17 patients were followed for more than 5 years, and two for 18 and 21 years, respectively. Hällen refers to the same series in 1969 by which time he has extended terms of follow-up by 3 years.

According to Hobbs (1969), follow-up periods of 5 years are required, occasionally even 10 years, " before a benign prognosis can be assumed "; Sleeper and Cawley (1969) declared that patients presenting with M-proteinaemia should be followed-up several times a year for 2 years, and thereafter once yearly.

According to the three case reports submitted here, it seems rather doubtful whether the above quoted follow-up periods actually were sufficiently long; it seems rather as if follow-up of this type of patient should never cease. Multiple myeloma may apparently develop after clinically asymptomatic phases of 24 years' standing. If the three patients concerned had died from another cause one year earlier, the diagnosis would have been established as one of " benign monoclonal gammopathy ".

For the time being we shall have to accept that multiple myeloma occasionally may run courses like those here described. It remains to be seen whether such courses are rare or of common occurrence; they may even be found to be the normal ones.

The above discussed case reports should be collated with those dealt with by Hobbs (1967, 1969, and 1971) and by Salmon and Smith (1970) in their studies of the preclinical development of multiple myeloma. According to these authors, the lesions had been preceded by long preclinical phases during which M-protein-aemia represented the exclusive symptom. This phase is preceded by another in which M-protein in serum actually may be present but only in concentrations too small to be detected. Throughout this protracted, symptom-free phase, the bone marrow will be a site of growth of myeloma cells.

The author wishes to express his gratitude to the Heads of the Departments to which the patients were admitted; the author is much obliged for their permission to have access to the case records concerned. Furthermore the author is indebted to Mr. Aksel Birch-Andersen, B.Eng., who in 1950–56 performed the electrophoreses according to Tiselius, and to Mr. Bendt Mansa, Graduate in Pharmacology, who in 1962–63 carried out the electrophoreses according to Tiselius and the immunoelectrophoreses.

### REFERENCES

Bichel, J.—(1964) *Acta. med. scand.*, **176**, 165.
Brücher, H.—(1970) *Schweiz. med. Wschr.*, **100**, 340.
Hällen, J.—(1966) *Acta med. scand.*, Suppl., 462.—(1969) *Nord. Med.*, **82**, 885.
Hobbs, J. R.—(1967) *Br. med. J.*, iii, 699.—(1969) *Proc. R. Soc. Med.*, **62**, 773.—(1971) *Br. med. J.*, ii, 67.
Kyle, R. A. and Bayrd, E. D.—(1966) *Am. J. Med.*, **40**, 426.
Noergaard, O.—(1964) *Acta med. scand.*, **176**, 137.—(1955) *Acta path. microbiol. scand.*, **37**, 329.—(1954) *Acta path. microbiol. scand.*, **34**, 336.
Salmon, S. and Smith, B. A.—(1970) *J. clin. Invest.*, **49**, 1114.
Sleeper, C. A. and Cawley, L. F.—(1969) *Am. J. clin. Path.*, **51**, 395.
Stevens, A. A.—(1965) *Archs intern. Med.*, **115**, 90.
Weicker, H., Brücker, H., Huhnstock, K., Meiser, J. and Plügge, H.—(1965) *Dt. Arch. klin. Med.*, **211**, 14.